# Synaptic potentiation of anterior cingulate cortex contributes to chronic pain of Parkinson’s disease

**DOI:** 10.1186/s13041-021-00870-y

**Published:** 2021-11-06

**Authors:** Zhaoxiang Zhou, Penghai Ye, Xu-Hui Li, Yuxiang Zhang, Muhang Li, Qi-Yu Chen, Jing-Shan Lu, Man Xue, Yanan Li, Weiqi Liu, Lin Lu, Wantong Shi, Ping-Yi Xu, Min Zhuo

**Affiliations:** 1 Center for Neuron and Disease, Frontier Institutes of Science and Technology, Xi’an Jiaotong University, Xi’an, 710049 China; 2 Institute of Brain Research, Qingdao International Academician Park, Qingdao, Shandong China; 3 Department of Physiology, Faculty of Medicine, University of Toronto, Medical Science Building, 1 King’s College Circle, Toronto, ON M5S 1A8 Canada; 4 Department of Neurology, The First Affiliated Hospital of Guangzhou Medical University, Guangzhou, China; 5 Department of Life Sciences, Graduate School of Arts and Sciences, University of Tokyo, Tokyo, Japan

**Keywords:** Parkinson’s disease, Chronic pain, ACC, LTP, MPTP

## Abstract

Parkinson’s disease (PD) is a multi-system neurodegenerative disorder. Patients with PD often suffer chronic pain. In the present study, we investigated motor, sensory and emotional changes in three different PD mice models. We found that 1-methyl-4-phenyl-1,2,3,6-tetrahydropyridine (MPTP)-treatment caused significant changes in all measurements. Mechanical hypersensitivity of PD model induced by MPTP peaked at 3 days and persisted for at least 14 days. Using Fos transgenic mice, we found that neurons in the anterior cingulate cortex (ACC) were activated after MPTP treatment. Inhibiting ACC by bilateral microinjection of muscimol significantly reduced mechanical hypersensitivity and anxiety-like responses. By contrast, MPTP induced motor deficit was not affected, indicating ACC activity is mostly responsible for sensory and emotional changes. We also investigated excitatory synaptic transmission and plasticity using brain slices of MPTP treated animals. While L-LTP was blocked or significantly reduced. E-LTP was not significantly affected in slices of MPTP treated animals. LTD induced by repetitive stimulation was not affected. Furthermore, we found that paired-pulse facilitation and spontaneous release of glutamate were also altered in MPTP treated animals, suggesting presynaptic enhancement of excitatory transmission in PD. Our results suggest that ACC synaptic transmission is enhanced in the animal model of PD, and cortical excitation may play important roles in PD related pain and anxiety.

## Introduction

Parkinson’s disease (PD) is a neurodegenerative disease classically characterized by motor dysfunction [1]. In addition to the motor symptoms, the non-motor symptoms of PD such as pain and anxiety, are gaining more clinical attention [2]. There are 30–95% of patients with PD suffering from different forms of pain, including acute pain and chronic pain [3]. More than 40% PD patients also suffer anxiety [4]. However, there is no effective clinical treatment for PD-related chronic pain and anxiety. Different animal models have been developed for the study of basic mechanism of PD, including 1-methyl-4-phenyl-1,2,3,6-tetrahydropyridine (MPTP)-treated model, 6-hydroxydopamine (6-OHDA)-treated model as well as A53T transgenic mice [5, 6].

Most of previous PD basic studies mainly focused on midbrain dopaminergic neurons [7], less is known about the ACC. The anterior cingulate cortex (ACC) is a key cortical region in pain perception, chronic pain and emotional anxiety and fear [8–10]. Excitatory synaptic transmission in the ACC undergo both pre- and post-synaptic long-term potentiation (LTP) in different animal models of chronic pain [11–13]. Furthermore, inhibiting or blocking ACC LTPs produced significantly reduction of behavioral sensory sensitization and injury-related anxiety [9, 14]. Previous studies from human brain imaging reported that ACC functions may be affected in PD patients [15, 16]. For example, Jonas et al. reported that some PD patients showed subtle gray matter atrophy in the ACC in compared to healthy control [16]. Moreover, positron emission tomography (PET) study showed that the turnover of dopamine is affected within the ACC in PD patients [15]. We recently proposed that ACC excitation may contribute to chronic pain in PD patients [17], however, no direct evidence has been reported. In the present study, we used different experimental approaches to investigate the roles of ACC in PD. Different behavioral tests were used to study the motor function, sensory sensitivity, and emotional anxiety in three PD mice models. Synaptic plasticity of ACC in PD conditioning was recorded by a multielectrode array recording system.

## Materials and methods

### Animals

Adult male mice (6 to 8 weeks) were used. C57BL/6 mice were purchased from the Experimental Animal Center of Xi’an Jiaotong University (Xi’an, China). Fos-GFP mice were obtained from The Jackson Laboratory (stock number: 014135). A53T transgenic mice were gifted by Zhaohui Liu (Medical School of Soochow University). Mouse line was maintained on a C57/BL6 background. Animals were maintained on a 12 h light/dark cycle with food and water provided ad libitum. All procedures and handling of animals were performed with permission according to the guidelines of Xi’an Jiaotong University.

### MPTP treatment

Mice were injected intraperitoneally (i.p.) with MPTP (20 mg/kg) or sterile saline solution (four times at 2 h intervals) [18].

### 6-OHDA injection

Under anesthesia by inhalation of isoflurane (1–3%), the animals were placed on a stereotaxic frame. Bilateral injections of 6-OHDA (4 μL of a 2 μg/μl in 0.2% ascorbic acid saline solution; each animal received a total of 16 μg of 6-OHDA injection, 8 μg at each side) was performed in the dorsal striatum (1 mm anterior to Bregma, 1.7 mm lateral from the midline, 2.9 mm beneath to the surface of the skull). 6-OHDA was injected manually at a rate of 1 μl/min. After the injection the needle was left in place for 3 min before slowly retracting it to prevent reflux. Control animals received the same volume of saline according to the same procedure. After surgery, the animals were allowed to recover for 2 weeks before starting the behavioral assessment [19].

### Microinjection into the ACC

Under anesthesia by inhalation of isoflurane (1–3%), 25-gauge guide cannulas were implanted bilaterally into the ACC (0.9 mm anterior to Bregma, 0.5 mm lateral from the midline, 1.4 mm beneath to the surface of the skull). Mice were given at least 2 weeks for recovery after cannula implantation. 30-gauge injection cannula was 0.8 mm lower than the guide. For intra-ACC infusion, 0.5 µl muscimol (1 µg/µl) or saline was delivered bilaterally within 90 s using a pump [20].

### Mechanical withdrawal threshold measurement

Mice were individually placed in a round, transparent container 20 cm in diameter and were allowed to acclimate for 30 min before testing. The mice paw withdrawal threshold was tested with von Frey filaments (Stoelting; Wood Dale, Illinois) applied to the paw. The animals were placed in Lucite cubicles over a wire mesh and acclimated for 30 min before testing. A series of filaments (0.008, 0.02, 0.04, 0.16, 0.4, 0.6, 1, 1.4, 2 g) with various bending forces (according to 0.078, 0.196, 0.392, 1.568, 3.92, 5.88, 9.8, 13.72, 19.6 mN) were applied to the plantar surface of the hindpaw until the mice withdrew from the stimulus. Each filament was applied twice. The lowest force at which a withdrawal response was obtained was then taken as the paw withdrawal threshold [13].

### Mechanical allodynia test

Mice were individually placed in a round, transparent container 20 cm in diameter and were allowed to acclimate for 30 min before testing. Mechanical sensitivity was assessed with a set of von Frey filaments. Based on preliminary experiments that characterized the threshold stimulus in untreated animals, the innocuous 0.04 g filament was used to detect mechanical allodynia. The filament was applied to the point of bending six times to the surfaces of the hindpaws. Positive responses consisted of prolonged hindpaw withdrawal followed by licking or scratching. Mechanical threshold was assessed on the basis of the responsiveness of the hindpaw to the application of von Frey filaments to the point of bending. The filament was applied over the dorsum of the paw while the animal was resting.

### Hot plate test

The mice were placed in the behavior room at least 0.5 h before the test to allow them to become accustomed to the experimental apparatus. The mouse was placed on the hot (55 ℃) plate and the latency to their first reaction (licking, shaking, jumping, or lifting of the hind paw) was recorded manually. If the mouse did not show any response within 20 s, the test was terminated to avoid tissue damage and the latency to the response was recorded as 20 s. Three values were used for average of latency to response.

### Tail flick test

The spinal nociceptive tail-flick reflex was evoked by focused, radiant heat provided by a 50 W projector lamp focused on a 1.5 mm by 10 mm area on the underside of the tail. The latency to reflexive removal of the tail from the heat was measured by a digital photocell timer to the nearest 0.1 s. The cutoff time of 10 s was used to minimize damage to the skin of the tail.

### Open-field test

To record locomotor activity, we used an open-field activity monitor (40 cm by 40 cm by 30.5 cm). This system uses paired sets of photo beams to detect movement in the open field, and movement is recorded as beam breaks. The open field is placed inside an isolation chamber with dim illumination and a fan. Locomotor activity was then measured for 30 min (20 cm * 20 cm for center zone, 40 cm * 40 cm for peripheral zone).

### Elevated plus maze

The elevated plus maze (EPM) consisted of two open arms and two closed arms situated perpendicular to each other. The maze was situated ~ 70 cm from the floor. For each test, mice were individually placed in the center square and allowed to move freely for 5 min. The number of entries and time spent in each arm were recorded. A video camera tracking system was used to generate the traces.

### Grip-strength test

The grip-strength apparatus is comprised of a wire grid connected to an isometric force transducer (dynamometer). In the grip-strength test, mice were held by their tails and allowed to grasp the grid with their forepaws. The mice were then gently pulled backward by the tail until the grid was released. The maximal force exerted by the mouse before losing grip was recorded. The mean of three measurements for each animal was calculated.

### RotaRod test

To test motor function, we used a RotaRod. The RotaRod test was performed by measuring the time each animal was able to maintain its balance while walking on a rotating drum. 1 h before testing, animals were trained on the RotaRod at a constant acceleration of 16 rpm until they could stay on for 30 s. For testing, the RotaRod was set to accelerate from 4 to 40 rpm over a 5 min period. Mice were given three trials with a maximum time of 300 s and a 5 min inter-trial rest interval. The latency to fall was taken as a measure of motor function.

### Homecage behaviors

24 h homecage behaviors were tested using AI homecage system (Shanghai Vanbi Intelligent Technology Co., Ltd.). The digital video cameras were mounted perpendicular to the cages. The cameras input into a Pelco video processor connected to computers. Video data were analyzed by Tracking Master software (Shanghai Vanbi Intelligent Technology Co., Ltd.). Activity time, standing time and hanging time were recorded and analyzed. During 24 h recording, mice were housed in standard cages on a 12 h light/dark cycle with food and water provided ad libitum [21].

### Immunohistochemistry

Mice were anaesthetized with isoflurane and perfused transcardially with PBS, followed by 4% paraformaldehyde in PBS. Brains were extracted from the skulls and incubated in 4% PFA at room temperature overnight. The brains were then removed, and postfixed in the same fixative for 4 h before cryoprotection in PBS containing 30% sucrose overnight at 4 °C. Brains were transferred to PBS and 30 µm coronal slices were taken using a vibratome and collected in PBS.

### Cell counting

Fluorescent images were acquired using light microscope (SLIDEVIEW VS200, Olympus, Tokyo, Japan) at the 20X objective. The laser with a wavelength of 488 nm was used for GFP excitation and 358 nm was used for DAPI. The number of Fos-GFP-positive, and DAPI-positive cells in a set region of interest (ROI) were quantified manually with ImageJ (https://imagej.nih.gov/ij/). The size of the ROI was standardized across brains, animals, experiments, and groups. All cell counting experiments were conducted blind to experimental group. To calculate the percentage of overlapping cells we counted the number Fos-GFP -positive cells and divided by the total number of DAPI-positive cells.

### Field potential recordings

#### Slice preparation

The general methods for preparing ACC slices were similar to those described previously [22]. Adult male mice were anesthetized by inhalation of isoflurane (1%-3%), and the whole brain was quickly removed from the skull and submerged in ice-cold oxygenated (95% O_2_ and 5% CO_2_) artificial cerebrospinal fluid (ACSF) containing the following (in mM): 124 NaCl, 4.4 KCl, 2 CaCl_2_, 1 MgSO_4_, 25 NaHCO_3_, 1 NaH_2_PO_4_, and 10 glucose, pH 7.35–7.45. After cooling in the ACSF for a short time, the whole brain was trimmed to yield an appropriate sample to glue onto the ice-cold stage of a vibrating tissue slicer (VT1200S, Leica). Coronal brain slices (300 µm), containing the ACC, were prepared after the corpus callosum connection. After cutting, slices were then incubated in a submerged recovery chamber with the ACSF, for at least 2 h at room temperature.

#### Preparation of the multi-electrode array

The MED64 recording system (Panasonic, Japan) was used for extracellular field potential recordings. There is an array of 64 planar microelectrodes in the MED64 probe (P515A, Panasonic, Japan), arranged in an 8 × 8 pattern, with an interpolar distance of 150 μm. The surface of the MED64 probe was treated with 0.1% polyethyleneimine in 25 mmol/L borate buffer (pH 8.4) overnight at room temperature before experiments. Then the surface of probe was rinsed three times with sterile distilled water [22, 23].

#### Electrophysiological recordings

After incubation, one slice containing the ACC was transferred to the prepared MED64 probe and perfused with oxygenated (95% O_2_ and 5% CO_2_) ACSF at 28–30 ℃ and maintained at a 2 ml/min flow rate. The slice was positioned on the MED64 probe in such a way that the different layers of the ACC were entirely covered by the whole array of the electrodes, and then a fine-mesh anchor was placed on the slice to ensure its stabilization during the experiments. One of the channels located in the layer V of the ACC was chosen as the stimulation site, from which the best synaptic responses can be induced in the surrounding recording channels. Slices were kept in the recording chamber for at least 1 h before the start of experiments. Bipolar constant current pulse stimulation (1–10 µA, 0.2 ms) was applied to the stimulation channel and the intensity was adjusted so that a half-maximal field excitatory postsynaptic potential (fEPSP) was elicited in the channels closest to the stimulation site. The channels with fEPSPs were considered as active channels and their fEPSPs responses were sampled every 1 min and averaged every 5 min. The parameter of “slope” indicated the averaged slope of each fEPSP recorded by activated channels. Stable baseline responses were first recorded until the baseline response variation is less than 5% in most of the active channels within 0.5 h [22, 23]. For LTP recordings, basal synaptic responses were stably recorded at least 30 min as a baseline, then a theta-burst stimulation (TBS, five trains of burst with four pulses at 100 Hz, at 200 ms interval; repeated five times at intervals of 10 s, with the same stimulation intensity and stimulation site with baseline) was given to induce stable LTP. LTP in a channel was defined if the potentiated synaptic response increased at least 20% of baseline during the entire recording period [24].

#### In vitro whole-cell patch-clamp recording

Experiments were performed in a recording chamber by using an Olympus BX51W1 microscope with infrared DIC optics for the visualization of whole-cell patch clamp recording. In the present study, Evoked EPSCs (eEPSCs) were recorded from the layer II/III neurons with an Axopatch 200B amplifier (Molecular Devices, CA), and the stimulations were delivered by a bipolar tungsten stimulating electrode placed in the layer V/VI of the ACC slices. Control test pulses were given every 30 s. The amplitudes of eEPSCs were adjusted to between 50 and 100 pA to obtain a baseline. Paired pulse stimulations with a 50 ms interpulse interval were given during the recordings. Stable baseline recordings of eEPSCs were obtained for 10 min before the induction of the LTP protocol. The recording pipettes (pyramidal neurons, 3–5 MΩ) were filled with a solution containing 145 mM K-gluconate, 5 mM NaCl, 1 mM MgCl2, 0.2 mM EGTA, 10 mM HEPES, 2 mM Mg-ATP, and 0.1 mM Na_3_-GTP (adjusted to pH 7.2 with KOH). Spontaneous EPSCs (sEPSCs) were recorded with the K-gluconate internal solution and holding − 60 mV. Picrotoxin (100 µM) was always present to block GABA_A_ receptor-mediated inhibitory synaptic currents in all experiments [25].

### Statistical analysis

Results were expressed as mean ± SEM. Statistical comparisons were performed with one-way ANOVA or two-way ANOVA and Student’s t test. In all cases, *p < 0.05 was considered statistically significant.

## Results

### PD models induced by MPTP, 6-OHDA, and A53T transgenic mice

**Fig. 1:**
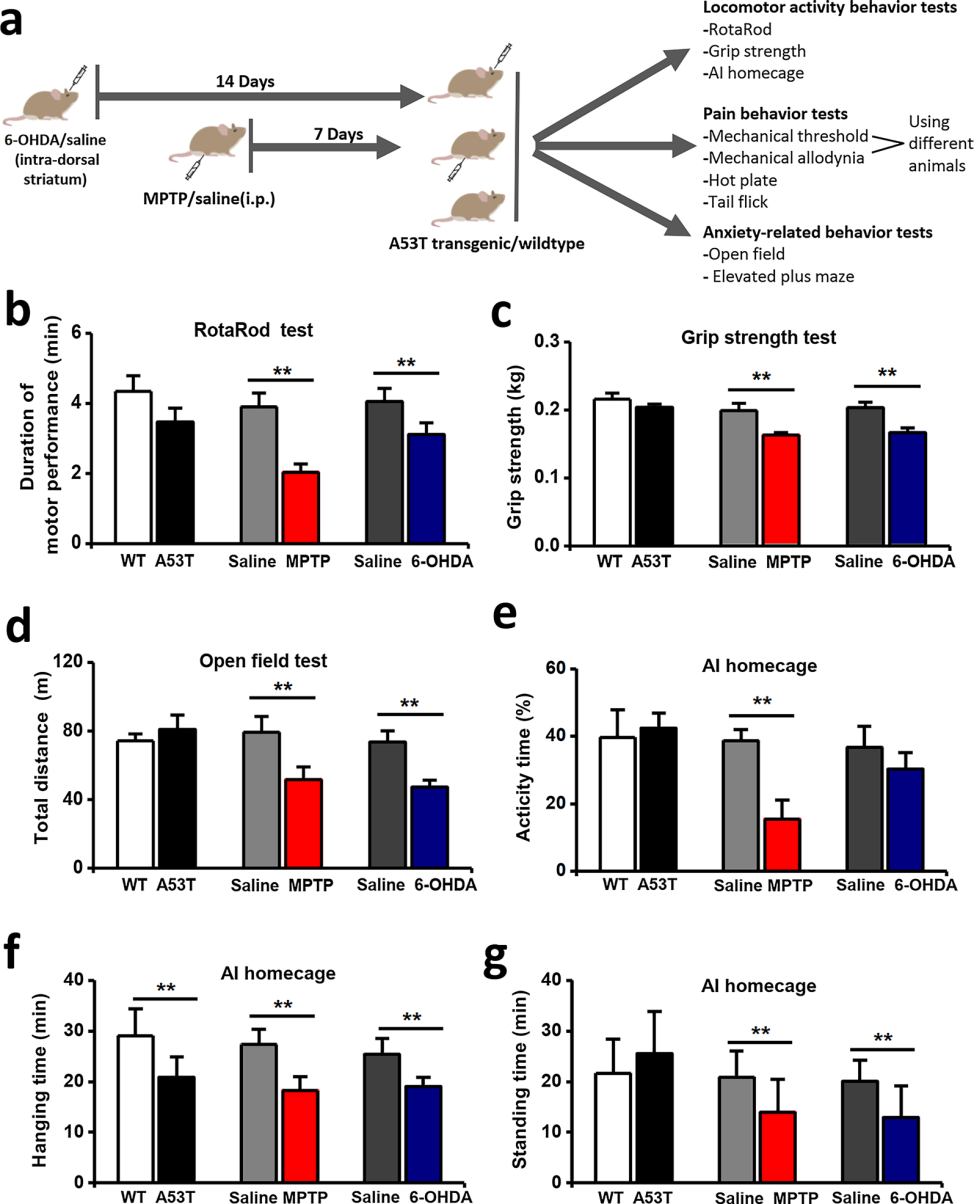
Motor function assessment of three PD mice models. **a** Diagram showing the experimental schedule and three PD mice models in this study. **b** Motor performance of MPTP-treated and 6-OHDA-treated mice were significantly decreased compared with saline-treated mice in the Rotarod test. **c** A severe grip strength deficit was observed in MPTP-treated mice group. **d** MPTP-treated and 6-OHDA-treated mice showed reduced movement distance in the open field test. **e**–**g** Homecage behavior test showed that MPTP-treated mice exhibited activity time (**e**), hanging time (**f**) were reduced in all three models, and MPTP-treated and 6-OHDA-treated mice showed reduced standing time (**g**) (n = 12 in WT and A53T groups, n = 13 in saline and MPTP groups, n = 12 in saline and 6-OHDA groups), **p < 0.01

Since the main symptoms of PD are motor deficits, we performed the RotaRod test, grip strength test, homecage behavior recording, and open field test, which are commonly used to confirm the phenotypes of PD model animals. The behavior tests data was listed in Table 1. Motor function was assessed by RotaRod test, grip strength test, and open field test, which significantly declined after MPTP or 6-OHDA administration, but not in A53T transgenic mice (Fig. 1a–-d). Moreover, in the homecage behavior test, only MPTP-treated mice showed reduced activity time (Fig. 1e). Standing time and hanging time were decreased in MPTP-treated and 6-OHDA-treated mice (Fig. 1f, g). A53T transgenic mice showed decreased hanging time, but not standing time (Fig. 1f, g). These results showed MPTP or 6-OHDA administration impaired the motor function of mice, including muscle capacity, the coordination of movement, and homecage behaviors.

**Table 1:** Behavior test results of motor function, pain, and anxiety-related behavior in A53T transgenic, MPTP, and 6-OHDA mice models used for the study of Parkinson’s disease

Behavior tests		A53T transgenic model		MPTP model		6-OHDA model	
		WT A53T		Saline MPTP		Saline 6-OHDA	
Motor function tests	Grip strength (kg)	0.22 ± 0.01 0.20 ± 0.01	N.S	0.20 ± 0.01 0.16 ± 0.004	↓**	0.20 ± 0.008 0.17 ± 0.007	↓**
	RotaRod (min)	4.34 ± 0.45 3.47 ± 0.40	N.S	3.90 ± 0.39 2.03 ± 0.24	↓**	4.06 ± 0.37 3.11 ± 0.33	↓**
	Open field -Total distance (m)	74.32 ± 4.10 80.88 ± 8.46	N.S	79.22 ± 9.23 51.66 ± 7.41	↓**	73.63 ± 6.46 47.23 ± 4.10	↓**
	AI homecage -Hanging time (min)	14.56 ± 2.63 10.41 ± 2.02	↓**	13.66 ± 1.54 9.11 ± 1.38	↓**	12.72 ± 1.56 9.54 ± 0.90	↓**
	AI homecage -Standing time (min)	10.82 ± 3.37 12.80 ± 4.14	N.S	10.46 ± 2.54 7.01 ± 3.22	↓**	10.07 ± 2.02 6.48 ± 3.11	↓**
	AI homecage -Activity time (%)	39.65 ± 6.20 42.37 ± 4.58	N.S	38.69 ± 3.30 15.36 ± 5.73	↓**	36.69 ± 2.37 35.36 ± 4.89	N.S
Pain behavior tests	Tail flick (s)	5.60 ± 0.37 5.00 ± 0.51	N.S	6.02 ± 0.26 4.23 ± 0.19	↓**	5.54 ± 0.46 4.20 ± 0.12	↓**
	Hot plate (s)	12.04 ± 1.09 10.09 ± 0.65	↓**	12.48 ± 0.96 10.08 ± 0.46	↓**	11.56 ± 0.81 10.10 ± 0.84	N.S
	Mechanical threshold (g)	0.73 ± 0.15 0.49 ± 0.11	↓**	0.84 ± 0.14 0.28 ± 0.05	↓**	0.76 ± 0.18 0.37 ± 0.10	↓**
	Mechanical allodynia (%)	5.00 ± 4.14 35.00 ± 9.69	↑**	3.5 ± 6.31 47.5 ± 12.08	↑**	6.67 ± 3.40 40.00 ± 18.76	↑**
Anxiety -related behavior tests	Open field -Time in central zone (%)	11.34 ± 3.20 13.64 ± 2.58	N.S	12.94 ± 2.30 4.27 ± 0.73	↓**	11.67 ± 2.37 4.35 ± 1.89	↓**
	Elevated plus maze -Time in open arms (%)	10.40 ± 2.09 11.94 ± 1.65	N.S	11.89 ± 0.86 4.59 ± 0.66	↓**	9.67 ± 0.81 5.7 ± 0.64	↓**
	Elevated plus maze -Number of entries	16.25 ± 4.20 14.65 ± 4.58	N.S	15.64 ± 3.30 12.96 ± 6.73	N.S	13.37 ± 2.37 11.38 ± 4.89	N.S

*N.S.*: not significant, ↓: decreased, ↑: increased

**p < 0.01

**Fig. 2:**
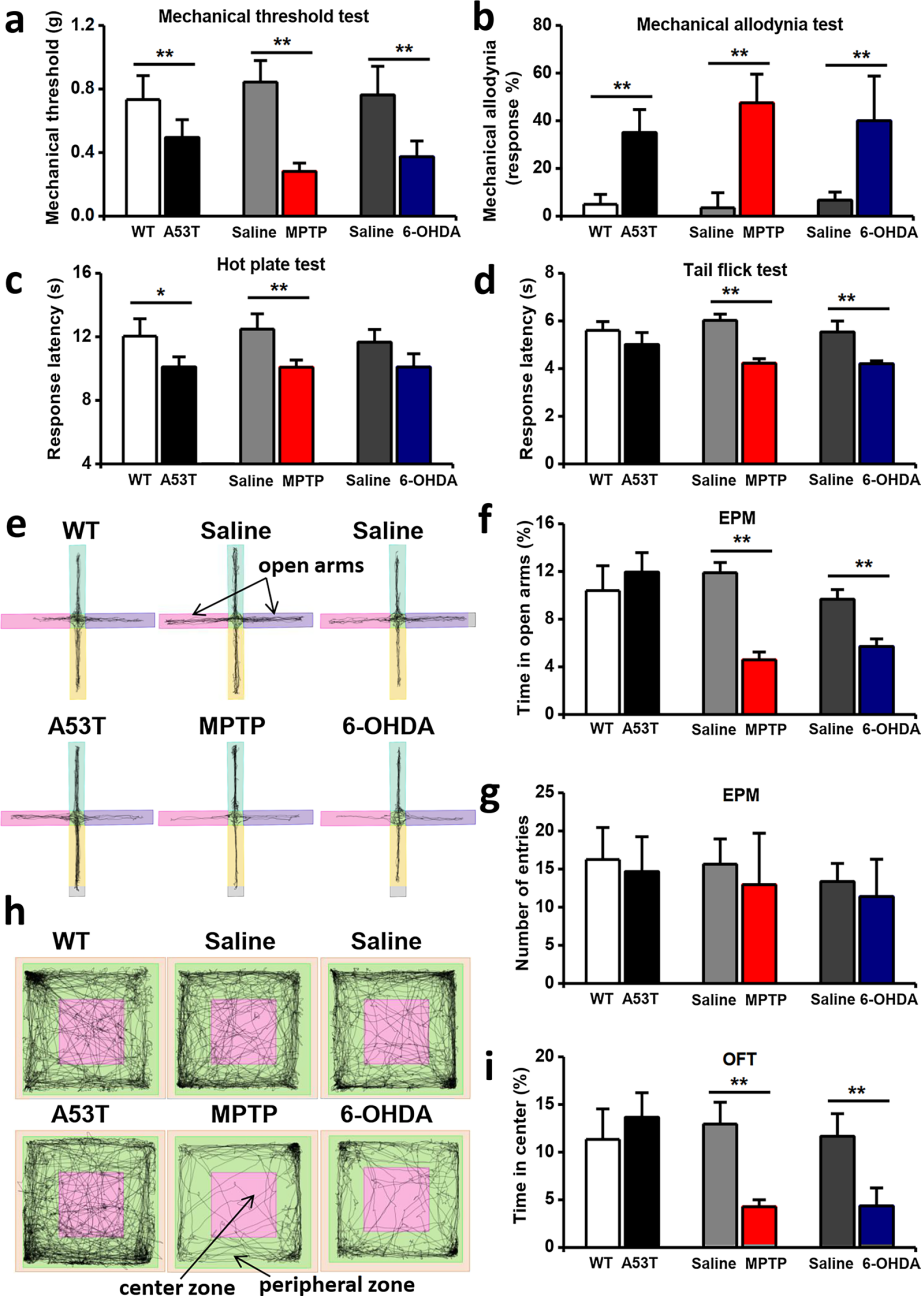
Pain and anxiety symptoms assessment of three PD mice models. **a**–**d** Mechanical threshold were reduced (**a**) and mechanical allodynia were increased (**b**) in all three PD models. Response latency in hot plate test was reduced in A53T transgenic and MPTP-treated mice (**c**). MPTP-treated and 6-OHDA-treated mice exhibited shorter response latency in tail flick test (**d**). **e–h** Representative traces showing the movement of mice in EPM (**e**) and open field test (**h**). **f**–**i** MPTP-treated and 6-OHDA-treated mice exhibited increased anxiety-related behaviors in EPM (**f**) and open field test (**i**), and there was no significant difference of entry number in EPM (**g**). (n = 10 in WT and A53T groups, n = 10 in saline and MPTP groups, n = 9 in saline and 6-OHDA groups) (In hot plate test, tail flick test, open field test and EPM, n = 12 in WT and A53T groups, n = 13 in saline and MPTP groups, n = 12 in saline and 6-OHDA groups. In mechanical threshold test, n = 6 in WT and A53T groups, n = 6 in saline and MPTP groups, n = 6 in saline and 6-OHDA groups. In mechanical allodynia test, n = 6 in WT and A53T groups, n = 7 in saline and MPTP groups, n = 6 in saline and 6-OHDA groups) *p < 0.05, **p < 0.01

Next, we performed EPM (Fig. 2e) and open field tests (Fig. 2h) to assess the anxiety behaviors in three PD models. Decreased time spent in open arms and distance in center area indicated that MPTP-treated and 6-OHDA-treated mice exhibited increased anxiety-related behaviors (Fig. 2f, i). However, there was no difference in time spent in open arms and time in center area between A53T transgenic mice and wildtype mice in the EPM and open field test (Fig. 2f, i). Taken together, these results indicate that the MPTP and 6-OHDA administration induces motor deficits and increased anxiety-related behaviors.

### Pain hypersensitivity

**Fig. 3:**
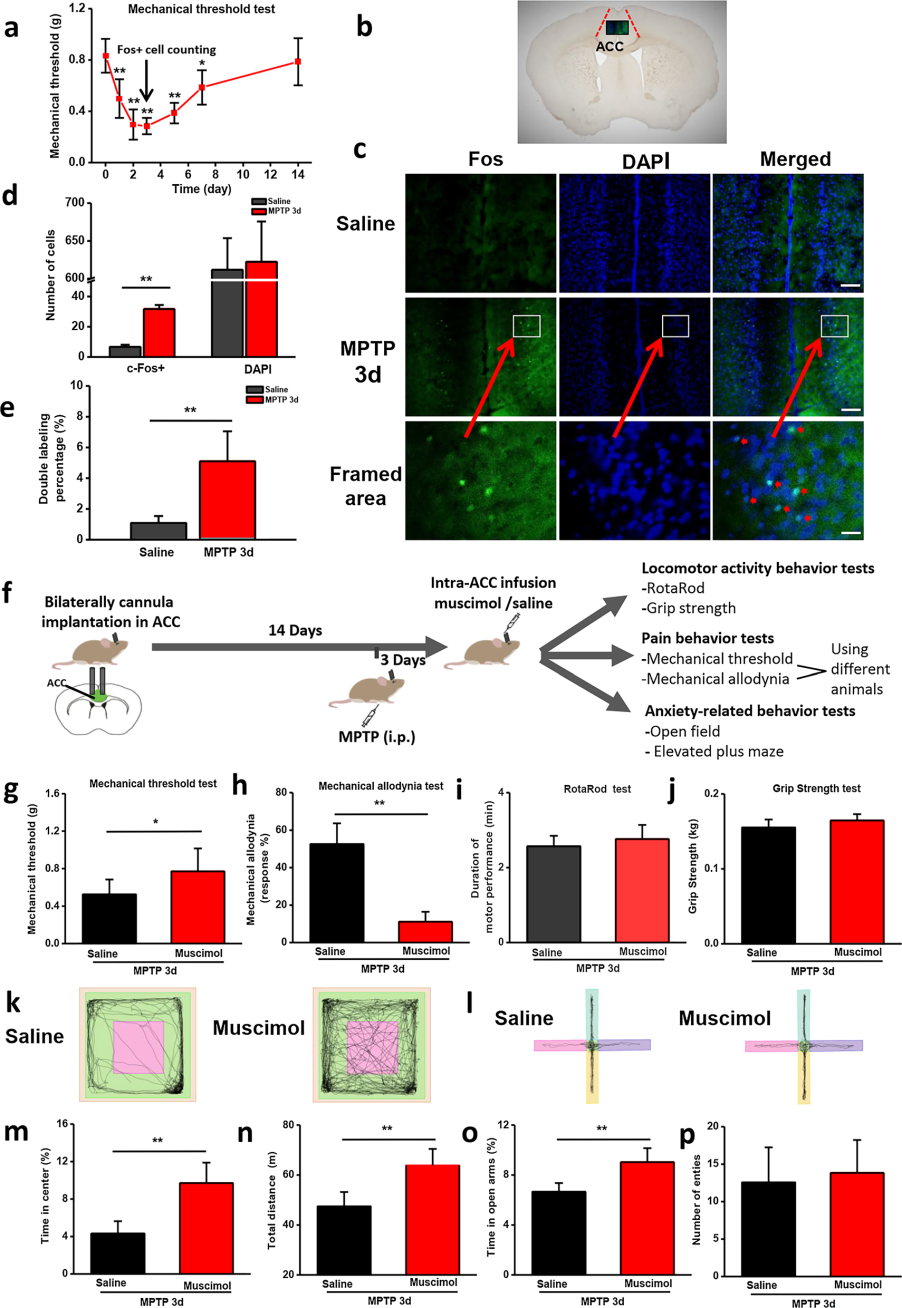
ACC neurons are involved in chronic pain and anxiety symptoms, but not motor function. **a** The temporal changes of mechanical threshold of MPTP-treated mice. **b** Diagram showing the location of ACC and the framed area, where the Fos-positive cells were counted. **c** Two sample slices of saline-treated and MPTP-treated 3d Fos-GFP mice. Bar = 250 μm in (**c**, top and middle), 50 μm in (**C,** bottom). **d**, **e** MPTP-treated mice (n = 5) showed a increased number of Fos-positive cells (**d**) and double labeling percentage (**e**) than saline-treated mice (n = 5). **f** Diagram showing the experimental schedule of bilateral injections of muscimol. (n = 7 in saline group, and n = 8 in muscimol group). Compared with saline group, mechanical threshold was increased (**g**) and mechanical allodynia was reduced (**h**) after injection of muscimol. However, there were no significant difference of the performance in RotaRod test (**i**) and grip strength test (**j**) between saline and muscimol groups. Representative traces showing the movement of mice in open field test (**k**) and EPM (**l**). There were significant decrease of anxiety-related behaviors in open field test (**m**) and EPM (**o**). The total distance of movement was increased in muscimol group than saline group (**n**). There was no significant difference of entry number in EPM (**p**). *p < 0.05, **p < 0.01

Chronic pain is the most prominent non-motor symptom in patients with Parkinson’s disease [26]. Therefore, we used several nociceptive behavioral tests to measure the mechanical allodynia, threshold, and thermal hyperalgesia. The behavior tests results were listed in Table 1. Compared to WT or saline group mice, the mice of three PD models exhibited reduced mechanical threshold and increased responses in mechanical allodynia test (Fig. 2a, b). In the thermal pain tests, A53T transgenic mice also showed reduced response time than WT group mice in only hot plate test, but not in tail flick test (Fig. 2c, d), and 6-OHDA-treated mice showed reduced response time than saline-treated mice in tail flick test, but not in hot plate test (Fig. 2c, d). However, MPTP-treated mice exhibited reduced response time in the both thermal threshold tests (Fig. 2c, d). Taken together, these three common PD models exhibited varying degrees of pain hypersensitivity. MPTP-treated mice model was used in next experiments. The pain hypersensitivity in MPTP-treated mice peaked at 3d and existed until 14d (Fig. 3a).

### Activation of ACC in MPTP induced PD model

The ACC is well known to be a critical region in sensory perception, especially in chronic pain [9]. However, whether ACC is involved in PD-related chronic pain remains unknown. To determine the role of the ACC in PD-related chronic pain, we utilized Fos-GFP mice that express the GFP in behaviorally activated Fos-expressing neurons, to assess the distribution of activated neurons in MPTP-treated PD mice. It has been shown that Fos could be used as a marker for neuronal activation following sexual stimulations [20]. To quantify neuronal activation in the ACC by PD-related chronic pain, we counted activated neurons (Fos-positive) and total neurons (DAPI-positive) in the ACC (Fig. 3a, b). We found that the number of activated neurons and double labeling percentage were significantly enhanced at 3 days after injection of MPTP (Fig. 3c–e).

### Inhibition of ACC reduces pain hypersensitivity and anxiety but not motor responses

Previous results found that ACC neurons were activated and the excitability of ACC neurons was enhanced in nerve injury mice [12, 27]. Therefore, we then performed pharmacological manipulations to see if the activation of the ACC is necessary to affect the behaviors in MPTP-induced PD mice (Fig. 3f). It is known that local injection of muscimol, a selective agonist for GABA_A_ receptors, could inactivate the ACC neuronal activity [20]. We found that bilateral injections of muscimol (0.5 μl per side) into the ACC of MPTP-treated mice significantly reduced mechanical allodynia and increased mechanical threshold (Fig. 3g, h). In grip strength and RotaRod tests, there was no difference between injection of muscimol and saline (Fig. 5i, j). Compared to saline group, both the time in open arms and time in center area were increased after injection of muscimol in the EPM and open filed tests (Fig. 3k–o). However, the total distance in open field test was increased (Fig. 3n).

### ACC LTP is occluded in MPTP-treated mice

**Fig. 4:**
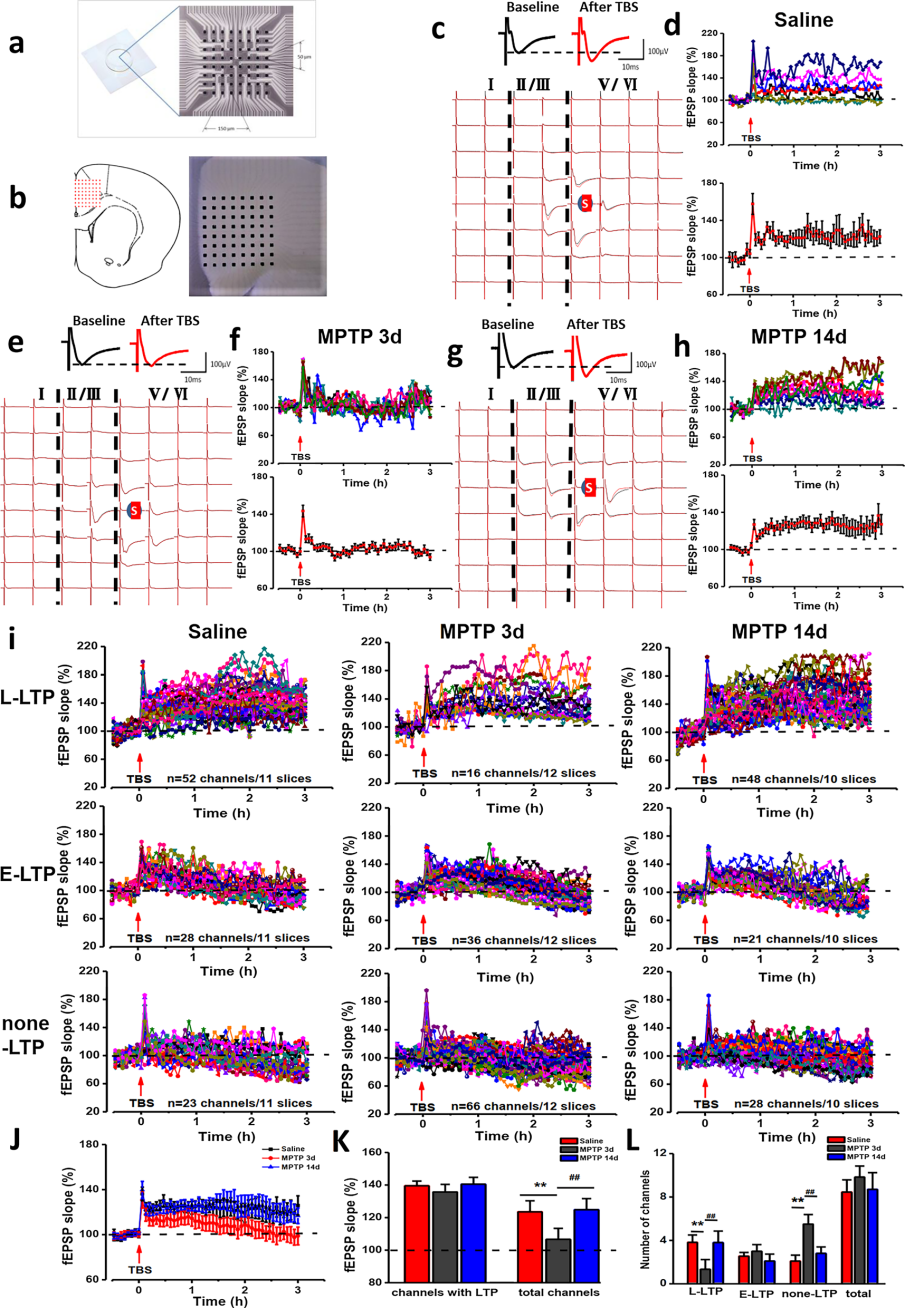
LTP recorded from slices of saline-treated, MPTP-treated 3d, and MPTP-treated 14d mice by extracellular field potential recording respectively. **a** Schematic diagram and the microphotograph showed the scale of the MED-64 probe (electrode size is 50 × 50 μm, interpolar distance of electrodes is 150 μm). **b** One example location of MED-64 probe on the ACC slice (left) and microscopy photograph of the location of ACC slice and MED-64 probe (right). Spatial distribution of extracellular field potential induced by electrical stimulation (marked as red circle) in layers V of the ACC slices from saline-treated (**c**), MPTP-treated 3d (**e**) and MPTP-treated 14d mice (**g**). The temporal changes of the fEPSP slopes of all traces (top) and averaged slope (bottom) of three ACC slices from saline-treated (**d**), MPTP-treated 3d (**f**) and MPTP-treated 14d mice (**h**). **i** All channels with L-LTP (top), E-LTP (middle) and none-LTP (bottom) were induced in saline-treated (left), MPTP-treated 3d (middle), and MPTP-treated 14d mice (right) (saline: 11 slices/7 mice; MPTP 3d: 12 slices/8 mice; MPTP 14d: 10 slices/7 mice). **j** The temporal changes of the averaged fEPSP slopes of all slices from saline-treated, MPTP-treated 3d, and MPTP-treated 14d mice. **k** MPTP-treated group showed reduced averaged fEPSP slope of all activated channels, but not all channels with LTP. **l** MPTP-treated 3d group showed reduced averaged number of LTP channels and increased none-LTP channels than saline-treated group or MPTP-treated 14d group. **p < 0.01, MPTP 3d vs saline. ##*p* < 0.01, MPTP 3d vs MPTP 14d

Increasing evidence suggests that LTP and LTD in the ACC, are causally related to chronic pain [8, 28]. Thus, we next investigated the LTP and LTD in the ACC using saline-treated and MPTP-treated mice. A 64-channel multielectrode array recording system (MED64) was used to record LTP and LTD in the ACC. We placed a slice containing the ACC on the MED64 chamber and ensured the recording microelectrodes covered the ACC area (Fig. 4a, b). One channel located in the deep layer of ACC was chosen as the stimulation site.

The fEPSP slopes of all activated channels were recorded from superficial to deep layer around the stimulation site for 0.5 h min as a baseline (Fig. 4c). After recording stable baseline for 0.5 h, we applied TBS to evoke network LTP in the ACC. In the ACC of saline-treated mice, TBS induced late-phase LTP (L-LTP) in most of the active channels, which was similar to our previous reports [23]. In one typical slice of saline-treated mice, the final averaged slope of all 8 channels from the slice was 128.9 ± 8.9% of the baseline at 3 h after applying TBS (Fig. 4d). In all 103 activated channels that recorded from 11 slices of 7 saline-treated mice, we found that there were 52 channels with L-LTP, 28 channels with E-LTP and 23 channels showed none-LTP (Fig. 4i). The final averaged fEPSP slope of all activated channels was 125.5 ± 6.8% of the baseline at 3 h in saline-treated mice and the final averaged fEPSP slope of all channels with L-LTP was 139.5 ± 2.9% of the baseline at 3 h (Fig. 4k).

Unlike the saline-treated mice (Fig. 4c, d), TBS failed to induce LTP in most of the active channels in the MPTP-treated mice (Fig. 4e). In one typical slice of MPTP-treated 3d mice, the final averaged slope of all 11 channels from the slice was 104.0 ± 10.4% of the baseline at 3 h after applying TBS (Fig. 4f). In all 118 activated channels that we recorded from 12 slices from 7 MPTP-treated 3d mice, we found that there were 16 channels with L-LTP, 36 channels with E-LTP and 66 channels showed none-LTP (Fig. 4i). The final averaged fEPSP slope of all activated channels was 106.6 ± 6.7% of the baseline at 3 h in MPTP-treated 3d mice (Fig. 4k).

However, after MPTP injection 14d, TBS successfully induced L-LTP in most of the active channels. In one typical slice of MPTP-treated 14d mice, the final averaged slope of all 10 channels from the slice was 125.4 ± 8.6% of the baseline at 3 h after applying TBS (Fig. 4g, h). In all 97 activated channels that we recorded from 10 slices from 6 MPTP-treated 14d mice, we found that there were 48 channels with L-LTP, 21 channels with E-LTP and 28 channels showed none-LTP (Fig. 4i). The final averaged fEPSP slope of all activated channels was 124.9 ± 6.8% of the baseline at 3 h in MPTP-treated 14d mice and the final averaged fEPSP slope of all channels with L-LTP was 140.5 ± 4.2% of the baseline at 3 h (Fig. 4k). The temporal changes of the averaged fEPSP slopes of all slices from three groups were showed in Fig. 4j.

Further analysis of the number of channels with L-LTP, E-LTP and none-LTP respectively showed that there were 3.9 ± 0.7 channels with L-LTP, 2.5 ± 0.4 channels with E-LTP, and 2.1 ± 0.6 channels with none-LTP in each slice of saline-treated mice on average (Fig. 4l). In MPTP-treated 3d mice, there were 1.3 ± 0.9 channels with L-LTP, 3 ± 0.6 channels with E-LTP and 5.5 ± 0.9 channels with none-LTP in each slice on average (Fig. 4l). And there were 3.9 ± 1.1 channels with L-LTP, 2.1 ± 0.5 channels with E-LTP and 2.8 ± 0.6 channels with none-LTP in each slice of MPTP-treated 14d mice on average (Fig. 4l). There was no statistical difference in the number of different types of total channels among saline-treated, MPTP-treated 3d and MPTP-treated 14d mice (Fig. 4l). However, the number of channels with L-LTP was significantly reduced in MPTP-treated 3d mice, but not MPTP-treated 14d mice (Fig. 4l). The number of channels with none-LTP was increased in MPTP-treated 3d mice, but not MPTP-treated 14d mice (Fig. 4l).These results indicate that the induction of LTP in ACC were occluded in most channels in MPTP-treated mice.

### Recruitment of synaptic responses is blocked in MPTP-treated mice

**Fig. 5:**
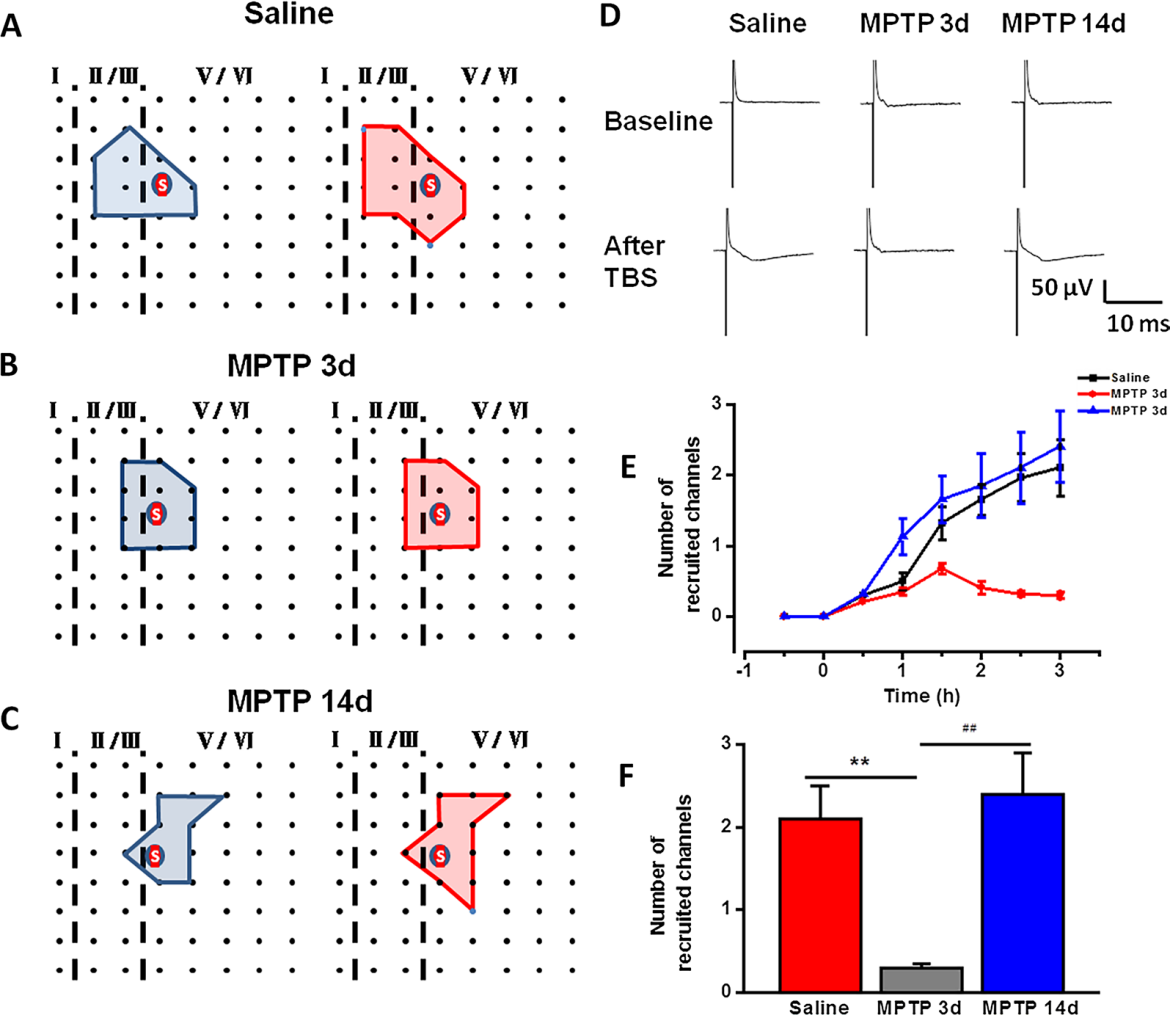
Recruited responses induced by TBS in the ACC of saline-treated, MPTP-treated 3d, and MPTP-treated 14d mice. **a**–**c** Basal activated area (blue) and recruited area (red) induced by TBS in typical slices from saline-treated (**a**), MPTP-treated 3d (**b**), and MPTP-treated 14d (**c**) mice respectively. The recruited channels were shown as blue dots. **d** Samples showed the recruited responses induced by TBS. **e** The temporal changes of the number of the recruited fEPSPs in saline-treated, MPTP-treated 3d, and MPTP-treated 14d mice. **f** The averaged number of recruited channels in saline-treated, MPTP-treated 3d, and MPTP-treated 14d mice (saline: 11 slices/7 mice; MPTP 3d: 12 slices/8 mice; MPTP 3d: 10 slices/7 mice). **p < 0.01, MPTP 3d vs saline. ##*p* < 0.01, MPTP 3d vs MPTP 14d

Previous studies showed that some silent responses were recruited after L-LTP induction [22, 23]. To test the effect of MPTP injection on the network recruitment in the ACC, we analyzed all recruited channels from saline-treated, MPTP-treated 3d, and MPTP-treated 14d mice respectively. In Fig. 5a, it showed a spatial distribution with recruited channels in a typical slice of saline-treated mice. The recruited channels mainly located on the edge of the activated area (Fig. 5a–c) and the amplitude which was about 0 μV during baseline would increase over time after applying TBS protocol (Fig. 5d). By analyzing the average number of the recruited channels from saline-treated (2.1 ± 0.4), MPTP-treated 3d (0.3 ± 0.05), and MPTP-treated 14d mice (2.4 ± 0.5) (Fig. 5f), we found that TBS cannot induce channel recruitment in MPTP-treated 3d mice ( Fig. 5b). The time course of the recruitment changes in three groups were shown in Fig. 5e. The number of recruited channels of saline-treated and MPTP-treated 14d mice gradually increased across the extended time scale after TBS induction, but not in MPTP-treated 3d mice. These results indicate that few channels were recruited after TBS in MPTP-treated mice.

### LTD in ACC is not affected in MPTP-treated mice

**Fig. 6:**
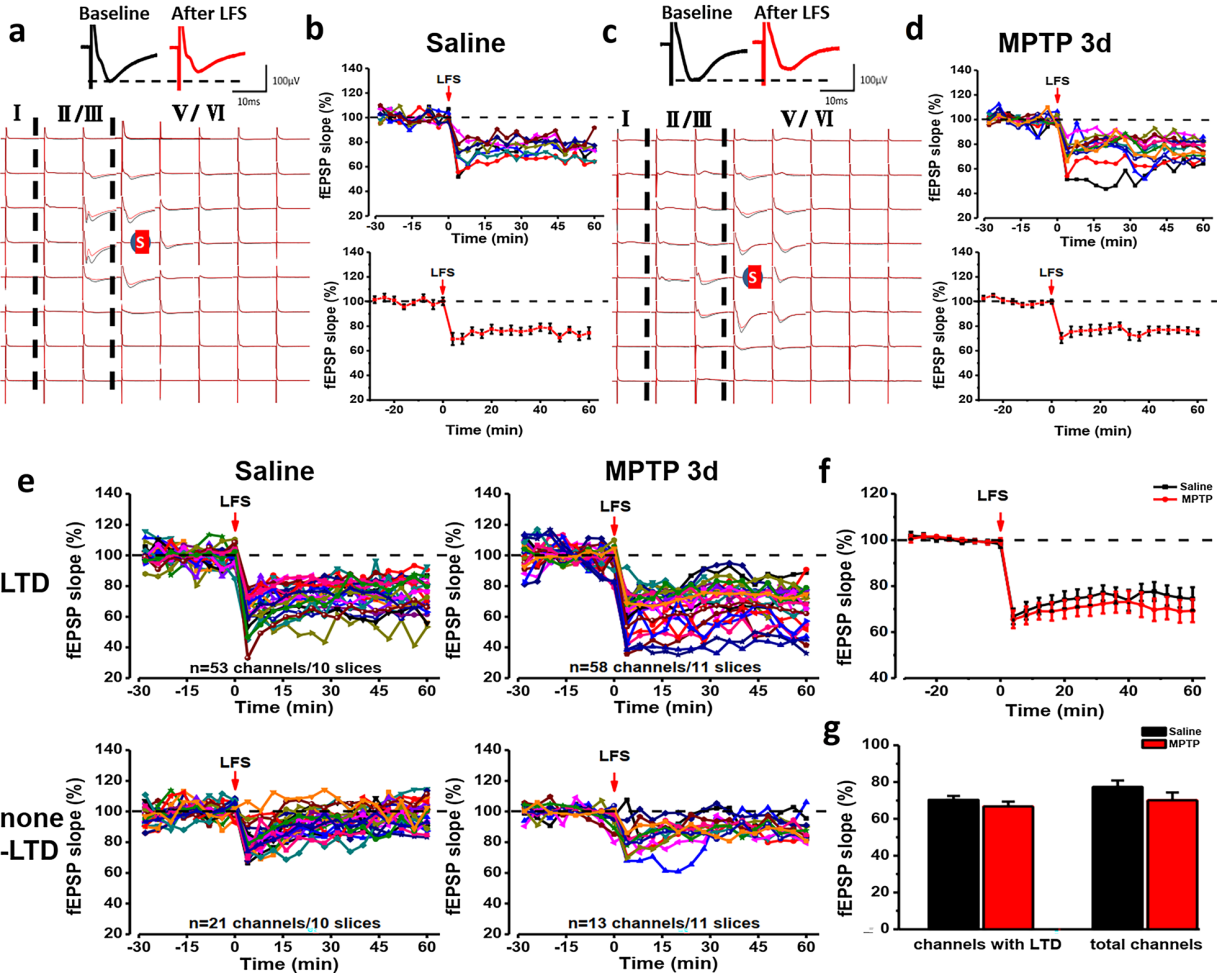
LTD recorded of ACC slices from saline-treated and MPTP-treated 3d mice by extracellular field potential recording respectively. Spatial distribution of extracellular field potential induced by electrical stimulation (marked as red circle) in layers V of the ACC slices from saline-treated (**a**) and MPTP-treated 3d (**c**). The temporal changes of the fEPSP slopes of all traces (top) and averaged slope (bottom) of two ACC slices from saline-treated (**b**) and MPTP-treated 3d (**d**). **e** All channels with LTD (top) and none-LTD (bottom) were induced in saline-treated (left), and MPTP-treated 3d mice (right) (saline: 10 slices/6 mice; MPTP 3d: 11 slices/6 mice). **f** The temporal changes of the averaged fEPSP slopes of all slices from saline-treated and MPTP-treated 3d mice. **g** There was no significant difference of averaged fEPSP slope of all activated channels and all channels with LTD between saline-treated and MPTP-treated mice

To explore the effect of MPTP injection on LTD in ACC, we recorded the fEPSPs baseline of all activated channels for 30 min before LTD inducing protocol was applied (LFS: 1 Hz, 900 pulses, with the same intensity as baseline recording). As shown in Fig. 6a–d, we got two different types of synaptic plasticity in all activated channels: channels with a slope decrease of more than 20% at 60 min represented LTD and channels that the fEPSP slope did not change after LFS represented none-LTD (Fig. 6c).

In a typical sample slice of a saline-treated mouse, there were 11 channels being activated, the final averaged slope of all 11 channels was 78.3 ± 6.6% of the baseline at 60 min after applying LFS (Fig. 6a, b). In a typical sample slice of a MPTP-treated 3d mouse, the final averaged slope of all 11 channels was 77.8 ± 7.1% of the baseline at 60 min after applying LFS (Fig. 6c, d). Next, we compared the final averaged slope of LTD in saline-treated and MPTP-treated 3d mice. There were 74 activated channels from 10 slices of 6 saline-treated mice and 71 activated channels from 11 slices of 6 MPTP-treated mice respectively (Fig. 6e). There was no difference in the final averaged slope of activated channels with LTD and all channels between MPTP-treated and saline-treated mice after applying LFS on stimulation site (Fig. 6f, g). These results suggested that intraperitoneal administration of MPTP didn’t affect LTD induced by LFS in the ACC.

**Fig. 7:**
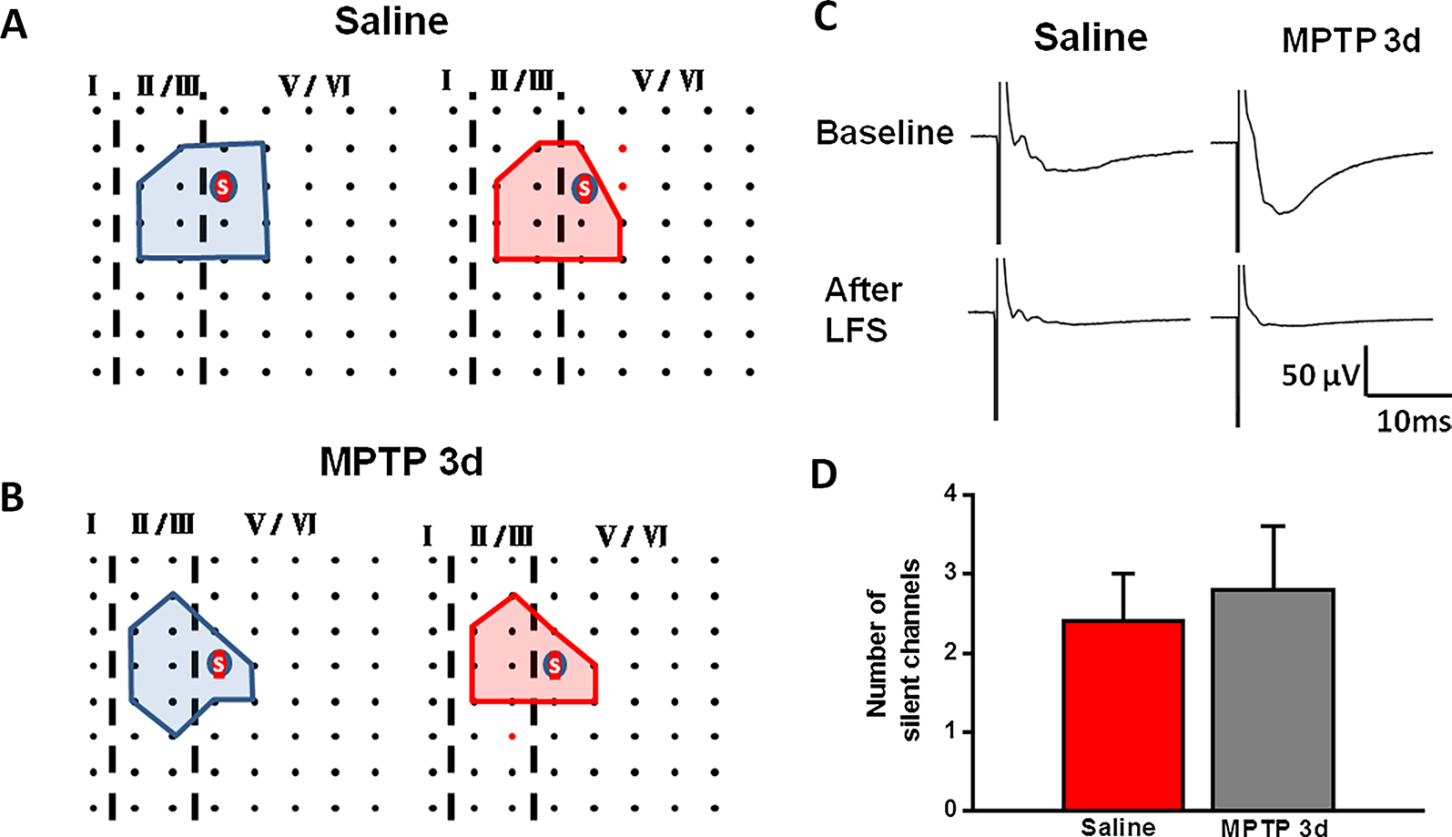
Activated channels were silenced by LFS in saline-treated and MPTP-treated 3d mice. **a**, **b** Basal activated area (blue) and silent area (red) induced by LFS in two typical slices from saline-treated (**a**) and MPTP-treated 3d (**b**) mice. The silent channels are shown as red dots. **c** The change of fEPSP amplitude of the silent channels in two typical slices from saline-treated and MPTP-treated 3d mice. **d** The average number of the silent channels in saline-treated mice and MPTP-treated 3d mice (saline: 10 slices/6 mice; MPTP 3d: 11 slices/7 mice)

There are hypotheses indicate that functional synapses can be transformed into silent synapses by endocytosis and inactivation of postsynaptic membrane α-amino-3-hydroxy-5-methyl-4-isoxazolepropionic acid (AMPA) receptor [29, 30]. In the present study, we found that some of active channels became silent after LFS (Fig. 7a, b). At 30 min after LFS, the amplitudes of the channels that were activated during baseline were decreased to about 0 μV (Fig. 7c). Next, we compared the number of silent channels that appeared after LFS in the ACC between saline-treated and MPTP-treated mice. There were 3.3 ± 1.5 silent channels in each slice of saline-treated mice on average and this result was similar to that in MPTP-treated mice (3.3 ± 0.8 silent channels, Fig. 7d). These results indicate that the transform of activated synapses to silent ones was not affected in MPTP-treated mice.

ACC神经元的兴奋性在慢性疼痛的过程中有重要意义, 为了检测帕金森模型小鼠的突触前兴奋性传递, 我们检测了ACC神经元的双脉冲刺激比值 (PPR) 。作为常常用来评估突触前功能的参数, 帕金森小鼠ACC神经元的PPF(1.33 ± 0.05) 显著低于对照组小鼠 (1.93 ± 0.14) 。

### Enhanced presynaptic transmission in the ACC in MPTP-treated mice

**Fig. 8:**
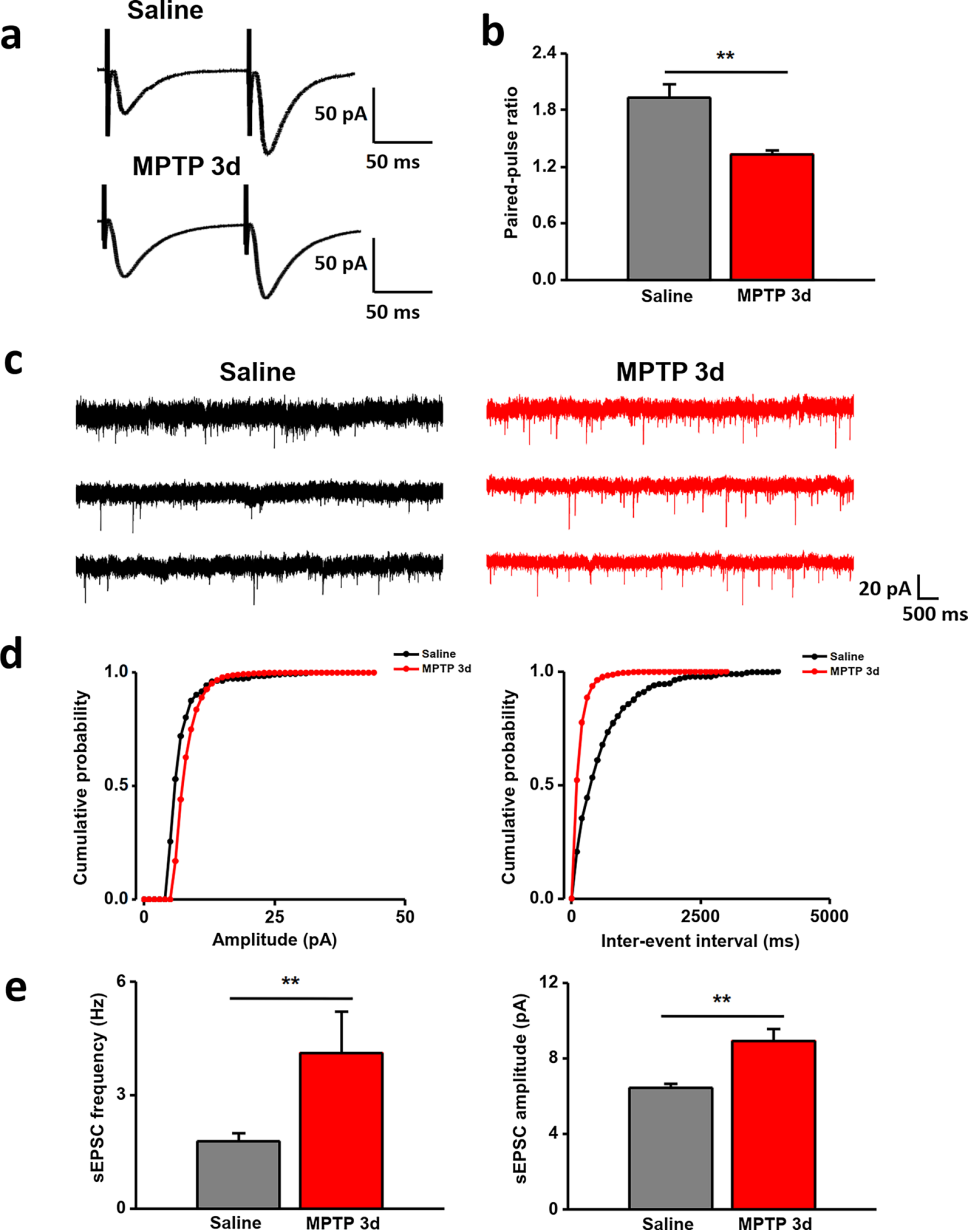
Enhanced presynaptic transmission in ACC. **a**, **b** Paired-pulse facilitation in ACC of PD model. **a** Representative traces of PPF with intervals of 50 ms. **b** PPF was reduced in in MPTP-treated mice compared with saline-treated mice. **c**–**e** sEPSCs recorded in the ACC of PD model. **c** Representative sEPSCs recorded in the ACC neuron in slices from saline-treated mice (left) and MPTP-treated mice (right). **d** Cumulative interevent interval (left) and amplitude (right) histograms of sEPSCs recorded in slices from saline-treated mice (black circles; n = 8 neurons) and MPTP –treated mice (red circles; n = 10 neurons). **e** Summary plots of sEPSC data. Averaged values of sEPSC parameters: mean peak frequency (left) and amplitude (right). **p < 0.01

To examine whether presynaptic mechanism is altered in the ACC of PD mice model, paired-pulse facilitation (PPF) was examined in ACC neurons in the present study. PPF is a transient form of plasticity commonly used as a measure of presynaptic function, in which the response to the second stimulus is enhanced as a result of residual calcium in the presynaptic terminal after the first stimulus. After MPTP treatment, there was a significant reduction in PPF in ACC neurons (n = 12 neurons/5 mice) compared with those from control mice (n = 10 neurons/4 mice) (Fig. 8a, b).

To further determine whether decreased PPF in the ACC in PD conditioning might be attributable to the increased presynaptic transmitter release probability, we recorded spontaneous EPSCs (sEPSCs) in ACC neurons. After MPTP treatment, there was an obvious increase of sEPSC frequency in ACC neurons compared with that of control group (saline: 1.8 ± 0.2 Hz, n = 9 neurons/4 mice; MPTP: 4.1 ± 1.1 Hz, n = 10 neurons/5 mice) (Fig. 2a–c). Furthermore, there was significant difference in the amplitude of sEPSCs between the two groups (saline: 6.5 ± 0.2 pA, n = 9 neurons/4 mice; MPTP: 8.9 ± 0.6 pA, n = 10 neurons/5 mice) (Fig. 8d, e). Together, these results indicate that the presynaptic transmission is enhanced in the ACC of MPTP-treated mice.

## Discussion

The present study, for the first time, revealed that the ACC is involved in PD-induced chronic pain in mice model. We found that ACC activation is required for chronic pain and anxiety symptoms in the animal model of PD, but not motor function. Moreover, LTP in ACC is occluded, and presynaptic neurotransmitter release is enhanced in MPTP-treated mice, suggesting long-term enhancement of both presynaptic and postsynaptic functions.

### Chronic pain symptom in different PD models

In our studies, we have used three PD models to offer insight into chronic pain in PD. In previous reports, MPTP-treated mice showed remarkably shorter nociceptive response latencies [31], and 6-OHDA-treated rats also exhibit reduced nociceptive thresholds [32]. Our results confirmed that MPTP-treated and 6-OHDA-treated mice models exhibited motor deficit and mechanical pain hypersensitivity. However, in thermal hyperalgesia tests, 6-OHDA-treated mice only showed decreased response latency in tail flick test, but not in hot plate test. Previous study observed thermal hyperalgesia in 6-OHDA-treated mice by using paw withdrawal latency to a radiant heat source [32]. This discrepancy might be attributable to the different methods used to test thermal pain threshold. The different methods to induce 6-OHDA model may also contribute to the difference of results. The normal or enhanced motor function in A53T transgenic model is pointed in previous studies [6, 33], but few study showed the pain hypersensitivity, which was observed in this study. The anxiety symptom in MPTP-treated and 6-OHDA-treated mice models is also consistent with previous studies [34, 35]. Besides obvious motor deficit, chronic pain and anxiety symptoms, we chose MPTP model in subsequent experiments since this model has specific advantages: (1) MPTP is the only known neurotoxin capable of causing degeneration of dopaminergic neurons in PD patients. (2) The injection of MPTP does not require any particular equipment or technical skills. (3) Systemic injection of MPTP produces a reliable and reproducible lesion of the nigrostriatal dopaminergic pathway. Therefore, MPTP-induced PD model is an appropriate model to understand the pain and anxiety symptoms study of PD patients.

### ACC is responsible for chronic pain and anxiety in PD

In this report, we found that ACC is activated bilaterally in MPTP-treated mice. By local infusion of muscimol, we found that inactivation of the ACC reversed the chronic pain and anxiety symptoms in PD, suggesting that ACC activity at least partially contribute to chronic pain and emotional anxiety in PD. Furthermore, we found that the motor impairment in MPTP-treated mice was not affected by ACC microinjection of muscimol, indicating that ACC is relatively selective involved in sensory and emotional functions.

### Synaptic plasticity in PD

LTP and LTD are two forms of synaptic plasticity that have been widely studied in learning and memory [36]. LTP is reported to be a key cellular model for cortical excitation in chronic pain [8, 9]. Our data suggest that the LTP in ACC was occluded during the persistent pain in PD model. It is similar with a synaptic plasticity study of nerve injury mice, which exhibited occlusion of LTP in ACC [13]. In the prefrontal cortex (PFC) of human patients and animal models of PD, LTP is also occluded [37, 38]. Another key finding is that the recruitment of cortical circuitry by LTP was also impaired MPTP-treated mice. In saline-treated mice, TBS induced recruitment of silent synapses in ACC, consistent with our previous studies [22]. However, there was few recruited channels in MPTP-treated mice, because LTP in ACC was occluded by enhanced pain responses. After MPTP-treated 14d, the recruitment responses recovered with the disappearance of LTP occlusion. These results strongly indicate that excitatory transmission in the ACC is potentiated in MPTP-treated mice. As another form of synaptic plasticity, LTD in ACC has been reported to be affected in chronic pain [28, 39, 40]. Wei et al. reported that amputation caused a loss of LTD in ACC that persisted for at least 2 weeks [40]. In this study, the unaltered LTD suggested that LTD in ACC is not involved in chronic pain in PD mice. Presynaptic synaptic plasticity has been identified as playing an important role in chronic pain and anxiety [12, 14, 25, 27]. Therefore, the presynaptic enhancement of neurotransmitter release in the ACC may contribute to the chronic pain and anxiety in mice of PD model.

### Potential drug target

Taken together, the presynaptic and postsynaptic excitatory transmission of ACC neurons are enhanced in MPTP-treated mice. The results are similar to ACC plastic changes reported in previous studies of chronic pain [12, 27]. As a novel target for chronic pain, adenylyl cyclase 1 (AC1) is essential for the presynaptic enhancement of glutamate release and the postsynaptic potentiation [12, 41]. Moreover, the AC1 inhibitor, NB001, produces powerful analgesic effects in different animal models of chronic pain [21, 42]. Our current findings strongly suggest that the AC1 inhibitor NB001 maybe used for the future treatment of chronic pain in PD patients.

## Conclusion

In summary, our results demonstrate that ACC plays an important role in PD related pain and anxiety, but not motor function. The discovery may help to understand the chronic pain symptom in PD. Further studies would investigate the cellular and synaptic mechanisms of PD-related chronic pain.

## Data Availability

The datasets used and analyzed during the current study are available from the corresponding author on reasonable request.

## Abbreviations

MPTP: 1-Methyl-4-phenyl-1,2,3,6-tetrahydropyridine
6-OHDA: 6-Hydroxydopamine
ACC: Anterior cingulate cortex
PD: Parkinson’s disease
LTP: Long-term potentiation
L-LTP: Late phase LTP
E-LTP: Early phase LTP
LTD: Long-term depression
EPM: Elevated plus maze
ACSF: Artificial cerebrospinal fluid
AMPA: α-Amino-3-hydroxy-5-methyl-4-isoxazolepropionic acid
AC1: Adenylyl cyclase 1
