# Correction to: Synaptic potentiation of anterior cingulate cortex contributes to chronic pain of Parkinson’s disease

**DOI:** 10.1186/s13041-022-00943-6

**Published:** 2022-06-22

**Authors:** Zhaoxiang Zhou, Penghai Ye, Xu-Hui Li, Yuxiang Zhang, Muhang Li, Qi-Yu Chen, Jing-Shan Lu, Man Xue, Yanan Li, Weiqi Liu, Lin Lu, Wantong Shi, Ping-Yi Xu, Min Zhuo

**Affiliations:** 1 Center for Neuron and Disease, Frontier Institutes of Science and Technology, Xi’an Jiaotong University, Xi’an, 710049 China; 2 Institute of Brain Research, Qingdao International Academician Park, Qingdao, Shandong China; 3 Department of Physiology, Faculty of Medicine, University of Toronto, Medical Science Building, 1 King’s College Circle, Toronto, ON M5S 1A8 Canada; 4 Department of Neurology, The First Affiliated Hospital of Guangzhou Medical University, Guangzhou, China; 5 Department of Life Sciences, Graduate School of Arts and Sciences, University of Tokyo, Tokyo, Japan

## Correction to: Mol Brain (2021) 14:161 https://doi.org/10.1186/s13041-021-00870-y

Following publication of the original article [1], the authors identified an incorrect paragraph with Chinese text at the end of the ‘Recruitment of synaptic responses is blocked in MPTP-treated mice’ subsection in the ‘Results’. This paragraph has been removed and the original article [1] has been updated.

## References

[1] Zhou Z, Ye P, Li X-H, Zhang Y, Li M, Chen Q-Y, Jing-Shan Lu, Xue M, Li Y, Liu W, Lin Lu, Shi W, Ping-Yi Xu, Zhuo M (2021). Synaptic potentiation of anterior cingulate cortex contributes to chronic pain of Parkinson’s disease. Mol Brain.

